# Tensor-Based Subspace Tracking for Time-Delay Estimation in GNSS Multi-Antenna Receivers

**DOI:** 10.3390/s19235076

**Published:** 2019-11-20

**Authors:** Caio C. R. Garcez, Daniel Valle de Lima, Ricardo Kehrle Miranda, Fábio Mendonça, João Paulo C. L. da Costa, André L. F. de Almeida, Rafael T. de Sousa

**Affiliations:** 1 Department of Electrical Engineering, University of Brasília, Brasília 70910-900, Brazil; daniel.vallelima@redes.unb.br (D.V.d.L.); fabio.mendonca@redes.unb.br (F.M.); joaopaulo.dacosta@ene.unb.br (J.P.C.L.d.C.);; 2 Department of Mechanical Engineering, University of Brasília, Brasília 70910-900, Brazil; ricardo.kehrle@redes.unb.br; 3 Department of Telefinformatics Engineering, Federal University of Ceará, Fortaleza 60455-760, Brazil; andre@gtel.ufc.br

**Keywords:** GNSS receivers, uniform linear array, time-delay estimation, tensor-based subspace estimation

## Abstract

Although Global Navigation Satellite Systems (GNSS) receivers currently achieve high accuracy when processing their geographic location under line of sight (LOS), multipath interference and noise degrades the accuracy considerably. In order to mitigate multipath interference, receivers based on multiple antennas became the focus of research and technological development. In this context, tensor-based approaches based on Parallel Factor Analysis (PARAFAC) models have been proposed in the literature, providing optimum performance. State-of-the-art techniques for antenna array based GNSS receivers compute singular value decomposition (SVD) for each new sample, implying into a high computational complexity, being, therefore, prohibitive for real-time applications. Therefore, in order to reduce the computational complexity of the parameter estimates, subspace tracking algorithms are essential. In this work, we propose a tensor-based subspace tracking framework to reduce the overall computational complexity of the highly accurate tensor-based time-delay estimation process.

## 1. Introduction

Global Navigation Satellite Systems (GNSS) provide geospatial positioning at any point in the gobe through the use of artificial satellites. These systems allow receptors on the surface of the Earth to determine geographic location by means of time of the transmitted signal time-delay, which is the interval that the signal takes to travel from the satellite to the receiver [1]. There are a wide number of applications for GNSS systems, e.g., civil aviation, navigation, geographic mapping, agricultural research, road flow, and defense. Additional recent applications of GNSS systems include: autonomous vehicles that require high safety and precision standards [2], real-time fishing vessel location for sustainable management of fisheries [3] and precision agriculture [4]. In the latter, the expensive agricultural machines are coordinated to work 24 hours a day and seven days a week and the costs with fertilization and the impact on the environment are drastically reduced due to the precise application of fertilizers.

The accurate positioning provided by GNSS receivers depend on its line of sight (LOS) with the satellites. In practice, non-LOS (NLOS) components are also present due to reflections on buildings, trees, and poles. These multipath components can drastically degrade positioning accuracy. In order to mitigate multipath components, several techniques using a single polarization antenna have been proposed in the literature, e.g., [5,6], but their capabilities are not sufficient for Safety-Critical Applications (SCA) or Liability-Critical Applications (LCA). Multi-antenna receivers have the capability to spatially separate the impinging signals [7,8], mitigating the effect of the multipath components and spatially filtering the LOS component. In this context, tensor-based approaches based on Parallel Factor Analysis (PARAFAC) models [9] have been developed, providing state-of-the-art performance. The state-of-the-art tensor-based multipath mitigation techniques applied to time-delay estimation are referred to as: the High Order Singular Value Decomposition based Time Delay Estimation (HOSVD/TDE) [10], the Direction of Arrival/Khatri-Rao Factorization (DoA/KRF) [11], the Semi-algebraic framework for approximate Canonical Polyadic Decompositions via Simultaneous Matrix Diagonalizations (SECSI) [12] and the Canonical Polyadic Decomposition by Generalized Eigenvalue Decomposition (CPD-GEVD) [13]. The tensor based approaches for GNSS receivers in [10,11] provide a high accuracy in comparison with the matrix based counterparts, but their current implementations are not suitable for real-time processing due to the high computational complexity of Singular Value Decomposition (SVD) for each sampling instant [14]. Therefore, an alternative to reduce the processing time of subspace estimations has been exploiting subspace tracking algorithms.

Subspace tracking methods are classified according to their approach and computational complexity. In case of computational performance, techniques are distinguished according to their complexity in relation to a straightforward SVD calculation. The direct-SVD method requires $O\{M^{3}\}$ operations, where *M* is the number of sensors on the antenna array. Improved high complexity algorithms like [15] achieve a complexity of $O\{M^{2}\cdot d\}$ operations by exploiting the model order *d*. The next category constitutes the medium complexity methods like [16] which have a computational cost of $O\{M\cdot d^{2}\}$ operations. Furthermore, the class of the low complexity methods provides O{M·d} operations and is considered as most suitable for real time implementation.

On the category of the approximation-based subspace methods, the Projection Approximation Subspace Tracking (PAST) algorithm introduced in [17] is a well-known low complexity scheme suitable for applications where only a rank-1 update on the estimated signal subspace matrix is necessary. In scenarios where the estimated subspace follows orthonormality or contains the exact basis of eigenvectors, low complexity methods such as the Fast Approximated Power Iteration (FAPI) [18] and the Fast Data Projection Method (FDPM) [19] are indicated. For more detailed information about the state-of-the-art subspace tracking algorithms, we refer the interested reader to [20].

Since the discussed subspace tracking techniques are matrix-based, approaches for tensor data have been developed [21]. The proposed methods are based on the relation between tensor decomposition models, such as PARAFAC [9] and Tucker [22], with matrix-based approaches. The objective is to track the tensor decomposition in an adaptive way while keeping the computational complexity at lower levels. On the adaptive setting for PARAFAC models, a framework for third-order tensors having one dimension growing with time has been proposed in [14,23]. In the adaptive Tucker decomposition approach, also referred to as dynamic tensor analysis [24], incremental tensor subspace learning [25] or tensor subspace tracking [21], subspace tracking methods are employed on the *n*-mode unfoldings of the tensor data in order to update the eigenspace estimates. For a more detailed literature review of advances in tensor-based subspace tracking, we refer to [26].

The main contribution of this paper is a tensor-based subspace tracking framework to update the signal subspace estimates without requiring SVD or High Order Singular Value Decomposition (HOSVD) computations at each time instant. As a consequence, we drastically reduce the computational complexity of the tensor-based time delay estimation of GNSS receivers. This paper is structured as follows: this first section includes the introduction along with the notation used in the paper. Section 2 presents the GNSS pre- and post-correlation data model. In Section 3, a tensor-based subspace tracking framework is proposed for the tensor-based time-delay estimation techniques. Section 4 presents the results of the Monte Carlo (MC) simulation and Section 5 draws the conclusion. A list of acronyms used along this paper can be found in Abbreviations.

### Notation

The notation used throughout this paper is organized as follows: scalars are denoted by lower-case letters: {a,b,c,…}, vectors are written as bold lower case letters: {a,b,c,…}, matrices by uppercase bold letters {A,B,C,…}, and tensors are represented as calligraphic letters: {A,B,C,…}. The superscripts: ${}^{T}$, ${}^{*}$, ${}^{H}$,${}^{-1}$ and ${}^{+}$, denote the transpose, conjugate, conjugate transpose (Hermitian), inverse, and pseudo-inverse of a matrix, respectively. For a matrix $A\in C^{M\times N}$, its *m*-th row is denoted by $A_{\left(m\;,\;:\right)}$ and its *n*-th column is denoted by $A_{\left(:\;,\;n\right)}$. The identity matrix is referred by the letter I. The Kronecker product is represented by ⊗ and the Khatri-Rao product (also known as column-wise Kronecker product) by ⋄. The Khatri-Rao product between matrices $A\in C^{I\times L}$ and $B\in C^{K\times L}$ is a matrix of dimensions IK×L. This product is defined as: $A⋄B=\left[\begin{array}{cccc} a_{1}⊗b_{1} & a_{2}⊗b_{2} & \cdots & a_{L}⊗b_{L} \end{array}\right]\in C^{IK\times L}$. The *n*-th mode unfolding of the tensor A is denoted by ${\left[A\right]}_{\left(n\right)}$ [27]. The n-mode product between a tensor A and a matrix B is denoted by $A\times_{\left(n\right)}B$.

## 2. Data Model

This section is divided in two parts. In Section 2.1 the tensor-based pre-correlation data model is explained. In Section 2.2 the post-correlation signal model constituted by the multiplication of the received signal by a compressed correlator bank is detailed. The data model used in this work is based on [10]. It is important to mention that in the scenario under consideration the model order $L_{d}$ should be known. Note that the estimation of the model order can be obtained using the approaches [28,29,30].

### 2.1. Pre-Correlation Signal Model

In the pre-correlation stage the *L* received signals for the Uniform Linear Array (ULA)-based GNSS receiver, composed of a LOS component and (L−1) delayed NLOS replicas, are temporally grouped into K epochs, for k=[1,2,…,K]. In each epoch, *N* samples are collected by the *M* elements of the array. Therefore, according to [10] the received signal can be modeled as:

(1) $$\begin{array}{c} X=I_{3,L_{d}}\times_{1}{\tilde{\Gamma }}^{T}\times_{2}{\tilde{C}}^{T}\times_{3}\tilde{A}+N\in C^{K\times N\times M}, \end{array}$$

where the matrix ${\tilde{\Gamma }}^{T}=\left[\begin{array}{ccc} \gamma_{1} & \cdots & \gamma_{\ell } \end{array}\right]\in C^{K\times L_{d}}$ contains the complex amplitudes related to each signal component of the *d*-th satellite along *K* epochs with $\gamma_{\ell }={\left[\begin{array}{ccc} \gamma [1] & \cdots & \gamma [K] \end{array}\right]}^{T}\in C^{K}$ being the vector with the complex amplitudes related to each epoch. The matrix $\tilde{A}=\left[\begin{array}{ccc} a(\phi_{1}) & \cdots & a(\phi_{\ell }) \end{array}\right]\in C^{M\times L_{d}}$ denotes the steering matrix of the *k*-th observation period of the *d*-th satellite where each $a(\phi_{\ell })$∈$C^{M}$ is referred as the steering vector with azimuth angle $\left(\phi_{\ell }\right)$. The matrix ${\tilde{C}}^{T}=\left[\begin{array}{ccc} c[t-\tau_{1}^{\left(d\right)}] & \cdots & c[t-\tau_{\ell }^{\left(d\right)}] \end{array}\right]\in R^{N\times L_{d}}$ contains the sampled and delayed PRN sequences of the *d*-th satellite. The term $I_{3,L_{d}}\in C^{L_{d}\times L_{d}\times L_{d}}$ is the third-order identity tensor and $N\in C^{K\times N\times M}$ refers to the AWGN characteristics of the channel which can be expressed as a tensor.

### 2.2. Post-Correlation Signal Model

As GNSS receivers perform a series of cross-correlations in order to align the incoming C/A code with a local generated replica, a bank of compressed correlators $Q_{\omega }^{\left(d\right)}=Q^{\left(d\right)}{\left(\Sigma V^{H}\right)}^{-1}\in C^{N\times Q}$ as presented in [10,31], is employed to multiply the received signal with all possible shifted replicas. This operation computes the cross-correlation vector necessary to perform the time-delay estimation. The application of the compressed correlator bank on the pre-processed signal in (1) leads to the following post-processed signal model:

(2) $$\begin{array}{cc} Y^{\left(d\right)} & =X\times_{2}{Q_{\omega }^{\left(d\right)}}^{T} \end{array}$$

(3) $$\begin{array}{cc} Y^{\left(d\right)} & =I_{3,L_{d}}\times_{1}\Gamma^{T}\times_{2}{\left({\mathrm{CQ}}_{\omega }^{\left(d\right)}\right)}^{T}\times_{3}A+N_{\omega }\in C^{K\times Q\times M}. \end{array}$$

## 3. Proposed Tensor-Based Subspace Tracking Framework

In this section, the proposed subspace tracking techniques for time-delay estimation are presented. First, the PAST–HOSVD/TDE is derived. Then, the PAST–DoA/KRF is formulated. Finally, computational complexity issues are discussed.

**Figure 1:**
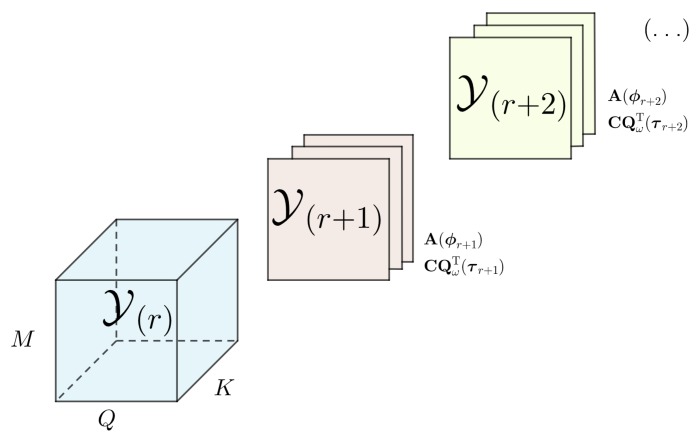
Observed epochs being concatenated into the dimension *K* of the previously acquired data.

As depicted in Figure 1, the adaptive tensor setting [26] which is also referred as online or incremental setting, is defined as a sequence of *R* observed tensors represented by $\left\{Y_{\left(r\right)}^{\left(d\right)}\right\}=\left\{Y_{\left(1\right)}^{\left(d\right)},\;\ldots ,\;Y_{\left(r\right)}^{\left(d\right)},\;\ldots ,\;Y_{\left(R\right)}^{\left(d\right)}\right\}$ serially acquired by means of their epochs. At a given time instant (t), each acquisition related to a single epoch, or to a collection *K* of epochs, contain their own parameters of time-delay $\left[\tau_{\left(r\right)}^{\left(d\right)}\right]$ and direction of arrival $\left[\phi_{\left(r\right)}^{\left(d\right)}\right]$ related to their corresponding factor matrices.

In accordance with (2) the expression for each tensor of the sequence $\left\{Y_{\left(r\right)}^{\left(d\right)}\right\}$ can be denoted as:

(4) $$Y_{\left(r\right)}^{\left(d\right)}=I_{3,L_{d}}\times_{1}\Gamma^{T}\left[r\right]\times_{2}{\left({\mathrm{CQ}}_{\omega }^{\left(d\right)}\right)}^{T}\left[\tau_{\left(r\right)}^{\left(d\right)}\right]\times_{3}A\left[\phi_{\left(r\right)}^{\left(d\right)}\right]+N_{\omega }\in C^{K\times Q\times M}.$$

In order to apply tensor-based subspace tracking methods, for the sake of simplicity only one epoch is considered in (4), implying into a matrix representation of the data associated to the reverse cyclical third-mode unfolding of $Y_{\left(r\right)}^{\left(d\right)}$ expressed as:

(5) $$\begin{array}{c} {\left[Y_{\left(r\right)}^{\left(d\right)}\right]}_{\left(3\right)}=A\left[\phi_{\left(r\right)}^{\left(d\right)}\right]{\left(\gamma \left[r\right]⋄{\left({\mathrm{CQ}}_{\omega }^{\left(d\right)}\right)}^{T}\left[\tau_{\left(r\right)}^{\left(d\right)}\right]\right)}^{T}\in C^{M\times Q}. \end{array}$$

### 3.1. PAST–HOSVD/TDE

According to [10], the HOSVD-based time-delay estimation (HOSVD/TDE) exploits the multidimensional structure of the received signal by decomposing it into principal singular vectors for each dimension [32]. Since the HOSVD/TDE is not an adaptive approach, a full HOSVD is computed for each new collected epoch, implying into a high computational complexity. In contrast to the state-of-the-art HOSVD-TDE, the proposed Projection Approximation Subspace Tracking (PAST)-HOSVD/TDE updates the multidimensional eigenspace by tracking a projection matrices of the different dimensions, allowing a considerable reduction of the computational complexity with similar accuracy.

The approach starts by separating the highly correlated signal components on each acquired snapshot. Therefore, the pre-processing techniques Forward-Backward Averaging (FBA) [7] and Expanded Spatial Smoothing (ESPS) [8] are applied on the reshaped received signal snapshot matrix ${\left[Y_{\left(r\right)}\right]}_{\left(3\right)}\in C^{M\times Q}$. These techniques exploit the symmetry of the antenna array in order to further improve the parameter estimation.

In order to apply FBA, two matrices $\Pi_{M}\in C^{M\times M}$ and $\Pi_{Q}\in C^{Q\times Q}$ are defined. The technique is applied according to the following equation:

(6) $$\begin{array}{c} Z_{\mathrm{FBA}}\;\left(r\right)=\left[\begin{array}{cc} {\left[Y_{\left(r\right)}^{\left(d\right)}\right]}_{\left(3\right)} & \Pi_{M}\;{\left[Y_{\left(r\right)}^{\left(d\right)}\right]}_{\left(3\right)}\;\Pi_{Q} \end{array}\right]\in C^{M\times 2Q}. \end{array}$$

The ESPS method is employed by means of selection matrices that generate $L_{s}$ subarrays with $M_{s}=M-L_{s}+1$ antenna elements. Each selection matrix is defined as:

(7) $$J_{\ell_{s}}=\left[\begin{array}{ccc} 0_{M_{s}\times \ell_{s}-1} & I_{M_{s}} & 0_{M_{s}\times L_{s}-\ell_{s}} \end{array}\right]\in C^{M_{s}\times \ell_{s}-1+M_{s}+L_{s}-\ell_{s}},$$

where $\ell_{s}=\left\{1,\cdots ,L_{s}\right\}$. Then SPS is applied to the FBA pre-possessed signal shown in Equation (6) according to the following method:

(8) $$W_{\mathrm{FBA}+\mathrm{SPS}}\;\left(r\right)=\left[\begin{array}{ccc} J_{1}Z_{\mathrm{FBA}}\left(r\right) & \cdots & J_{L_{s}}Z_{\mathrm{FBA}}\left(r\right) \end{array}\right]\in C^{M_{s}\times 2L_{s}\times Q}.$$

By applying the FBA and ESPS, the original antenna array dimension is split into three dimensions, the first one related to the reduced antenna arrays ($M_{s}$), the second related to the subarrays ($L_{s}$) and the third related to the FBA doubling the array aperture, implying into a dimension of size 2 [33,34]. Note that, bringing back the epoch dimension, $W_{\mathrm{FBA}+\mathrm{SPS}}\;\left(r\right)$ in (8) can be reshaped into a fourth order tensor $Z_{\mathrm{FBA}+\mathrm{ESPS}}\;\left(r\right)\in C^{2\times Q\times M_{s}\times L_{s}}$ which is referred to as the data tensor observed at each new snapshot [35]. Notice that $Z_{\mathrm{FBA}+\mathrm{ESPS}}\left(r\right)$ is a pre-processed and rearranged version of ${\left[Y_{\left(r\right)}\right]}_{\left(3\right)}\in C^{M\times Q}$ in (5). For futher information about ESPS and FBA applied to GNSS, we refer to [36].

Subsequently, the multidimensional filtering proposed in [10] is performed on $Z_{\mathrm{FBA}+\mathrm{ESPS}}\;\left(r\right)$, which HOSVD is given by:

(9) $$Z_{\mathrm{FBA}+\mathrm{ESPS}}\;\left(r\right)=G\times_{1}U^{\left(1\right)}\times_{2}U^{\left(2\right)}\times_{3}U^{\left(3\right)}\times_{4}U^{\left(4\right)}\in C^{2\times Q\times M_{s}\times L_{s}},$$

where $G\in C^{2\times Q\times M_{s}\times L_{s}}$ is defined as the core tensor and $U^{\left(1\right)}\in C^{2\times 2}$, $U^{\left(2\right)}\in C^{Q\times Q}$, $U^{\left(3\right)}\in C^{M_{s}\times M_{s}}$ and $U^{\left(4\right)}\in C^{L_{s}\times L_{s}}$ are unitary matrices containing the respective singular vectors $u^{\left(n\right)}$ of each *n*-mode unfolding of each mode of $Z_{\mathrm{FBA}+\mathrm{ESPS}}\;\left(r\right)$.

Since the signal model states that the LOS component has the greatest power when compared with the NLOS replicas, the *n*-mode singular vectors $u^{\left(n\right)}$ are ordered in the unitary matrices $U^{\left(n\right)}$ in a decreasing order of the magnitude of its corresponding singular values. Hence, the dominant singular vectors of the first, third and fourth modes of $Z_{\mathrm{FBA}+\mathrm{ESPS}}\;\left(r\right)$ in Equation (9) will be mostly correlated to the LOS component. The eigenfiltering process proposed in [10] is performed according to:

(10) $$q_{\mathrm{FBA}+\mathrm{ESPS}}\;\left(r\right)=\left(\begin{array}{c} Z_{\mathrm{FBA}+\mathrm{ESPS}}\;\left(r\right)\times_{1}{\left(u^{\left(1\right)}\right)}^{H}\times_{3}{\left(u^{\left(3\right)}\right)}^{H}\times_{4}{\left(u^{\left(4\right)}\right)}^{H} \end{array}\right)\Sigma V^{H}\in C^{1\times Q},$$

where the term $\Sigma V^{H}\in C^{Q\times Q}$, which multiplies the filter, compensates the correlation process with the bank shown in Equation (2). The output represents the multi-dimensional filtered cross-correlation values at each tap of the correlator bank. Later, the amount of points of the output of the correlator bank is increased via a cubic spline interpolation and one dimension peak search is performed in order to locate the time-delay of the greatest power component.

In order to obtain an estimation of each *n*-mode singular vector $u^{\left(n\right)}$ at time instant (t+1), PAST–HOSVD/TDE employs the tensor-based version of PAST proposed by [21]. The difference lies in the fact that the subspace tracking algorithm will update the *n*-mode singular vectors by tracking a projection matrix for the first, third and fourth modes of $Z_{\mathrm{FBA}+\mathrm{ESPS}}\;\left(r+1\right)\in C^{2K\times Q\times M_{s}\times L_{s}}$. The pseudo-code of the proposed technique can be consulted in Algorithm 1. The matrices ${\hat{U}}_{S}^{\left(1\right)}\left(0\right)=I_{2K\times L}$, ${\hat{U}}_{S}^{\left(3\right)}\left(0\right)=I_{M_{s}\times L}$, ${\hat{U}}_{S}^{\left(4\right)}\left(0\right)=I_{L_{s}\times L}$ refer to the signal subspace basis related to the respective first, third and fourth modes of $Z_{\mathrm{FBA}+\mathrm{ESPS}}\;\left(r\right)$ in (9) and ${\hat{C}}_{\mathrm{yy}}^{\left(1\right)}\left(0\right)={\hat{C}}_{\mathrm{yy}}^{\left(3\right)}\left(0\right)={\hat{C}}_{\mathrm{yy}}^{\left(4\right)}\left(0\right)=I_{L}$ is a d×d correlation matrix of the projection matrix Y(t) which is recursively updated in order to track the signal subspace associated to the corresponding unfolding of $Z_{\mathrm{FBA}+\mathrm{ESPS}}\;\left(r\right)$ at a given time instant (t+1). After obtaining an estimation for the signal subspace basis, the dominant eigenvectors are selected to perform the eigenfiltering process seen in (10). Note that the tensor-based version of PAST can run on the unfoldings of $Z_{\mathrm{FBA}+\mathrm{ESPS}}\;\left(r\right)$ in parallel, which provides an efficient implementation.

### 3.2. PAST–DoA/KRF

Since the post-correlation received signal model in Equation (1) follows a PARAFAC model, DoA/KRF proposed in [11] first estimates the factor matrices and then uses one factor matrix to estimate the time-delay. The approach starts by reconstructing the array steering factor matrix by means of a DoA estimation method referred as the Estimation of Signal Parameters via Rotational Invariance Techniques (ESPRIT) [37]. The remaining factor matrices are then obtained by means of the Khatri-Rao factorization (KRF). Later, the time-delay estimation is performed via the signal’s greatest power approach, as discussed on the previous section. The pseudo-code of DoA/KRF is provided in Algorithm A1.

When considering an adaptive tensor setting for real-time applications, DoA/KRF [11] updates the signal subspace estimation by performing a truncated SVD on each observed epoch or on a *K* collection period of epochs. In this context, to provide a reduction on the overall computational complexity, the proposed PAST–DoA/KRF method links the Tensor-based PAST [35] algorithm to the DoA/KRF time-delay estimation technique by providing a subspace basis to ESPRIT at each sampling instant. The pseudo-code of the proposed technique can be consulted in Algorithm 2. Notice that the tensor-based PAST technique consists of a rank-1 update of the estimated signal subspace matrix. The method does not provide the exact basis of eigenvectors related to the signal subspace of the covariance matrix of the observed data, but a mere basis to span it. Hence, since any basis belonging to $span(U_{s})$ is a solution for the shift invariance equation of ESPRIT [37], the array steering factor matrix can be rebuild by extracting information about given signal subspace estimation related to the antenna array dimension. We refer to [38] for more detailed information about ESPRIT applied to the estimated factor matrices in order to estimate the DoA.

### 3.3. Computational Complexity

In this assessment, the operation counts on the study of the computational complexity of the algorithms are expressed in terms of Multiply Accumulate (MAC) operations, referred as FLoating point OPeration (FLOP) counts [39]. Similarly as considered in [10], the objective of the computational complexity evaluation is to provide an assessment about the relative complexity difference between each algorithm. Since the proposed techniques differ from the state-of-the-art only when it concerns the update of the signal subspace estimation, operations such as the unfolding and the inverse-unfolding are not taken into account. In addition, the stage of finishing the correlation by multiplying the solution vector by $\Sigma V^{H}\in C^{Q\times Q}$ will also be ignored once it is an operation performed by all algorithms. For additional information concerning the absolute computational complexity of the state-of-the-art algorithms, we refer to [12].

It can be noticed from Table 1 that if the receiver decides to increase the number *K* of collected epochs, the computational cost of the proposed methods remains unaltered while the complexity of the state-of-the-art techniques increase.

**Algorithm 1:** PAST–HOSVD time-delay estimation.
Initialization.
${\hat{U}}_{s}^{\left(1\right)}\left(0\right)=I_{2\times L}$, ${\hat{U}}_{s}^{\left(3\right)}\left(0\right)=I_{M_{s}\times L}$; ${\hat{U}}_{s}^{\left(4\right)}\left(0\right)=I_{L_{s}\times L}$${\hat{C}}_{\mathrm{yy}}^{\left(1\right)}\left(0\right)={\hat{C}}_{\mathrm{yy}}^{\left(3\right)}\left(0\right)={\hat{C}}_{\mathrm{yy}}^{\left(4\right)}\left(0\right)=I_{L}$Section: Tensor-based PAST [21].fort=0,1,…do:*% – Tracking the signal subspace related to the dimension K*.$Y^{\left(1\right)}\left(t+1\right)={\hat{U}}_{s}^{\left(1\right)}{\;}^{\;H}\left(t\right)\;{\left[Z_{\mathrm{FBA}+\mathrm{ESPS}}\;\left(r\right)\right]}_{\left(1\right)}\left(t+1\right)$$C_{\mathrm{yy}}^{\left(1\right)}\left(t+1\right)=\beta \;C_{\mathrm{yy}}^{\left(1\right)}\left(t\right)+Y^{\left(1\right)}\left(t+1\right)Y^{\left(1\right)}H\left(t+1\right)$$G^{\left(1\right)}\left(t+1\right)={C_{\mathrm{yy}}^{\left(1\right)}}^{-1}\left(t+1\right)Y^{\left(1\right)}\left(t+1\right)$$E^{\left(1\right)}\left(t+1\right)={\left[Z_{\mathrm{FBA}+\mathrm{ESPS}}\;\left(r\right)\right]}_{\left(1\right)}\left(t+1\right)-{\hat{U}}_{s}^{\left(1\right)}\left(t\right)\;Y^{\left(1\right)}\left(t+1\right)$${\hat{U}}_{s}^{\left(1\right)}\left(t+1\right)={\hat{U}}_{s}^{\left(1\right)}\left(t\right)+E^{\left(1\right)}\left(t+1\right)\;{G^{\left(1\right)}}^{H}\left(t+1\right)$  *% – Tracking the signal subspace related to the dimension $M_{s}$*.$Y^{\left(3\right)}\left(t+1\right)={\hat{U}}_{s}^{\left(3\right)}{\;}^{\;H}\left(t\right)\;{\left[Z_{\mathrm{FBA}+\mathrm{ESPS}}\;\left(r\right)\right]}_{\left(3\right)}\left(t+1\right)$$C_{\mathrm{yy}}^{\left(3\right)}\left(t+1\right)=\beta \;C_{\mathrm{yy}}^{\left(3\right)}\left(t\right)+Y^{\left(3\right)}\left(t+1\right)Y^{\left(3\right)}H\left(t+1\right)$$G^{\left(3\right)}\left(t+1\right)={C_{\mathrm{yy}}^{\left(3\right)}}^{-1}\left(t+1\right)Y^{\left(3\right)}\left(t+1\right)$$E^{\left(3\right)}\left(t+1\right)={\left[Z_{\mathrm{FBA}+\mathrm{ESPS}}\;\left(r\right)\right]}_{\left(3\right)}\left(t+1\right)-{\hat{U}}_{s}^{\left(3\right)}\left(t\right)\;Y^{\left(3\right)}\left(t+1\right)$${\hat{U}}_{s}^{\left(3\right)}\left(t+1\right)={\hat{U}}_{s}^{\left(3\right)}\left(t\right)+E^{\left(3\right)}\left(t+1\right)\;{G^{\left(3\right)}}^{H}\left(t+1\right)$  *% – Tracking the signal subspace related to the dimension $L_{s}$*.$Y^{\left(4\right)}\left(t+1\right)={\hat{U}}_{s}^{\left(4\right)}{\;}^{\;H}\left(t\right)\;{\left[Z_{\mathrm{FBA}+\mathrm{ESPS}}\;\left(r\right)\right]}_{\left(4\right)}\left(t+1\right)$$C_{\mathrm{yy}}^{\left(4\right)}\left(t+1\right)=\beta \;C_{\mathrm{yy}}^{\left(4\right)}\left(t\right)+Y^{\left(4\right)}\left(t+1\right)Y^{\left(4\right)}H\left(t+1\right)$$G^{\left(4\right)}\left(t+1\right)={C_{\mathrm{yy}}^{\left(4\right)}}^{-1}\left(t+1\right)Y^{\left(4\right)}\left(t+1\right)$$E^{\left(4\right)}\left(t+1\right)={\left[Z_{\mathrm{FBA}+\mathrm{ESPS}}\;\left(r\right)\right]}_{\left(4\right)}\left(t+1\right)-{\hat{U}}_{s}^{\left(4\right)}\left(t\right)\;Y^{\left(4\right)}\left(t+1\right)$${\hat{U}}_{s}^{\left(4\right)}\left(t+1\right)={\hat{U}}_{s}^{\left(4\right)}\left(t\right)+E^{\left(4\right)}\left(t+1\right)\;{G^{\left(4\right)}}^{H}\left(t+1\right)$Eigenfiltering section [10].
 Select the dominant singular vectors of each ${\hat{U}}_{s}\left(t+1\right)$ previously estimated. Then apply the filtering process detailed in (10). $q_{\mathrm{FBA}+\mathrm{ESPS}}\;\left(r\right)=\left(\begin{array}{c} Z_{\mathrm{FBA}+\mathrm{ESPS}}\;\left(r\right)\times_{1}{\left({\hat{u}}^{\left(1\right)}\right)}^{H}\times_{3}{\left({\hat{u}}^{\left(3\right)}\right)}^{H}\times_{4}{\left({\hat{u}}^{\left(4\right)}\right)}^{H} \end{array}\right)\Sigma V^{H}\in C^{Q\times 1}$
end


**Algorithm 2:** PAST–DoA/KRF time-delay estimation.
Initialization.${\hat{U}}_{s}\left(0\right)=I_{M\times L}$Section: Tensor-based PAST [21].for t=1,2,…do:$Y\left(t+1\right)={\hat{U}}_{s}^{H}\left(t\right){\left[Y_{\left(r\right)}\right]}_{\left(3\right)}\left(t+1\right)$$C_{\mathrm{yy}}\left(t+1\right)=\beta \;C_{\mathrm{yy}}\left(t\right)+Y\left(t+1\right)Y^{H}\left(t+1\right)$$G\left(t+1\right)=C_{\mathrm{yy}}^{-1}\left(t+1\right)Y\left(t+1\right)$$E\left(t+1\right)={\left[Y_{\left(r\right)}\right]}_{\left(3\right)}\left(t+1\right)-{\hat{U}}_{s}\left(t\right)\;Y\left(t+1\right)$${\hat{U}}_{s}\left(t+1\right)={\hat{U}}_{s}\left(t+1\right)+E\left(t+1\right)\;G^{H}\left(t+1\right)$Section DoA/KRF [11]. Algorithm A1. 3.1 When performing subspace-based DoA methods, employ ${\hat{U}}_{s}\left(t\right)$ as an estimated basis, instead of performing a full EVD/SVD on the array covariance matrix.end


## 4. Simulations

Similarly to [10,11] we consider a scenario with a left center-hermitian uniform linear array receiver with M=8 elements and half-wavelength Δ=λ/2 spacing. Note that the GALANT hardware proposed by the German Aerospace Center (DLR) in [40] allows up to 16 antennas. Typical range goes from 4 to 6 antennas. The GNSS signal is a GPS second generation course acquisition PRN code from d=1 satellite at a carrier frequency $f_{c}=1575.42$ MHz, bandwidth B=1.023 MHz and chip duration $T_{c}=1/B=977.52$ ns with N=2046 samples collected every *k*-th observation period during K=30 epochs. Each epoch has duration Δt=1 ms. The number of impinging signals on the receiver is L=2, one LOS component with time-delay $\tau_{(\mathrm{LOS}})$ and one NLOS multipath replica with time-delay $\tau_{\left(\mathrm{NLOS}\right)}$, such that $\tau_{\left(\mathrm{NLOS}\right)}=\tau_{\left(\mathrm{LOS}\right)}+\Delta \tau$, where Δτ is the delay difference between each component. Their azimuth angle difference is $\Delta \phi =30^{{}^{\circ}}$. For the pre-processing techniques SPS/ESPS the array is divided into $L_{s}=5$ subarrays with $M_{s}=4$ elements each. Signal phases are independent and identically distributed ∼U[0,2π]. The number of correlators in the bank is Q=11 equally spaced **between** $-T_{c}$ and $T_{c}$. The carrier-to-noise ratio is $C/N_{0}=48$ dB-Hz, resulting in a pre-correlation ${\mathrm{SNR}}_{\mathrm{pre}}=C/N_{0}-10\log_{10}\left(2B\right)\approx -15.11$ dB. Post-correlation ${\mathrm{SNR}}_{\mathrm{post}}={\mathrm{SNR}}_{\mathrm{pre}}+G\approx 15$ dB and signal to multipath ratio (SMR) of 5 dB. For the tracking scenario, we consider a total of N=50 received tensors. The step variation of the angle of arrival between each acquired tensor is $\Delta \phi_{n}=0.25{}^{\circ}$. For the Tensor-based PAST subspace tracker, the forgetting factor $\beta_{\mathrm{PAST}}$ is set to 0.95.

The results are obtained by performing $M_{c}=1000$ Monte Carlo (MC) simulations to plot the root mean squared error (RMSE) expressed in meters, which is a measurement of the estimated time-delay multiplied by the speed of light, c=299792458 m/s. The expression is written as:

(11) $$\begin{array}{c} \mathrm{RMSE}\;\left(m\right)=c\;\sqrt{\frac{1}{M_{c}}\sum_{i=1}^{Mc}{\left(\tau_{i}-{\hat{\tau }}_{i}\right)}^{2}} \end{array}$$

**Figure 2:**
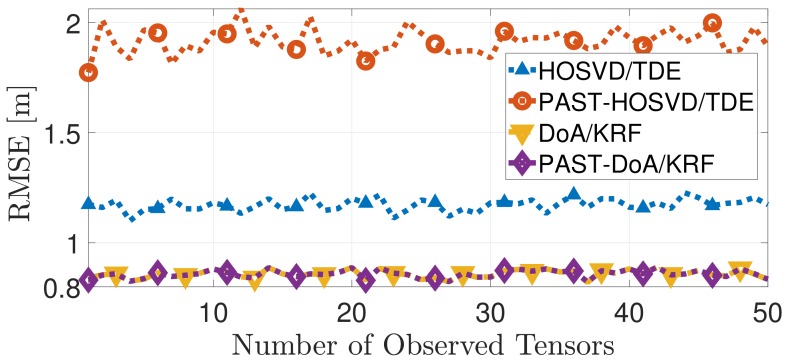
Comparison between the proposed techniques PAST–DoA/KRF and PAST-HOSVD/TDE and the state-of-the-art techniques DoA/KRF [11] HOSVD/TDE [10] in terms of RMSE.

The RMSE ($\tau_{\mathrm{LOS}}$) [m] for the techniques DoA/KRF, PAST–DoA/KRF, HOSVD/TDE and PAST–HOSVD/TDE simulated in the tracking scenario with the parameters described before is presented in Figure 2. Notice that PAST-DoA/KRF and DoA/KRF have similar performance while HOSVD/TDE and PAST–HOSVD/TDE present an average RMSE difference of 0.75 meters.

**Figure 3:**
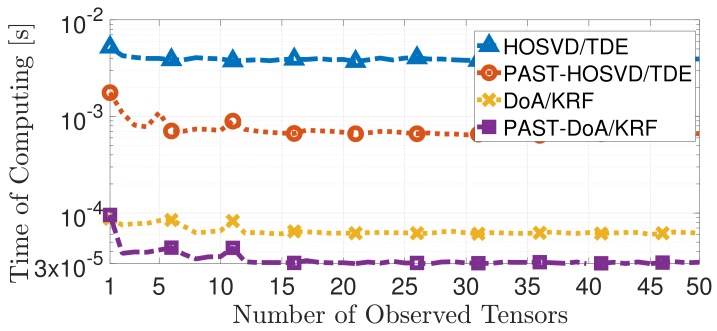
Comparison between the proposed techniques PAST–DoA/KRF and PAST-HOSVD/TDE and the state-of-the-art techniques DoA/KRF [11] HOSVD/TDE [10] in terms of Time of Computing.

On the computational cost of the simulation, notice in Figure 3 that PAST–DoA/KRF presents a lower computational cost when compared to DoA/KRF. The same is observed for PAST–HOSVD/TDE and HOSVD/TDE. The results correspond to the theoretical computational complexity discussed in Section 3.3.

**Figure 4:**
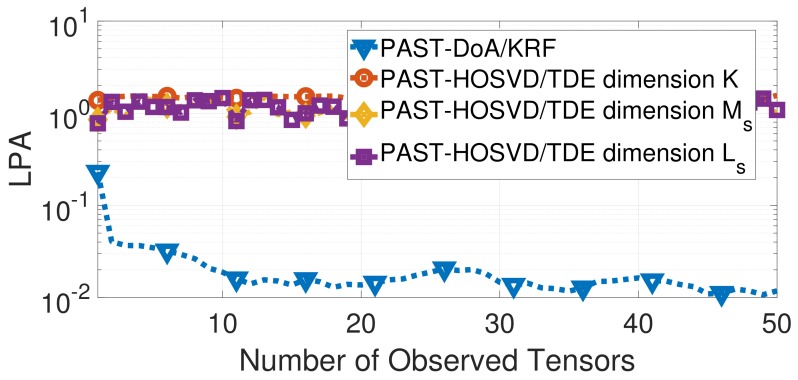
Comparison between the techniques PAST–DoA/KRF and PAST-HOSVD/TDE in terms of LPA.

In order to evaluate the subspace estimates provided by the proposed techniques, Figure 4 displays the Largest Principle Angle (LPA) between the true subspace and the estimated signal subspace along the number of observed tensors. The LPA between the column spaces of two matrices $U_{1}\;,\;U_{2}\in C^{M\times d}$ is computed based on [39] according to:

(12) $$\begin{array}{c} \mathrm{LPA}\;\left(r\right)=\cos^{-1}\left(\sigma_{\min}\left\{\mathrm{orth}{\left\{U_{1}\left(r\right)\right\}}^{H}\cdot \mathrm{orth}\left\{U_{2}\left(r\right)\right\}\right\}\right), \end{array}$$

where orth{·} is the orthonormal basis operator and $\sigma_{\min}\left\{\cdot \right\}$ is the smallest singular value operator.

Notice that the LPA of PAST–DoA/KRF is smaller than the LPA of the estimated basis of PAST–HOSVD/TDE related to the modes of the observed tensor. This difference reflects on the performance of the RMSE∼[m] as observed in Figure 2.

## 5. Conclusions

In this work a tensor-based subspace tracking framework for time-delay estimation in GNSS receivers is proposed. The proposed PAST-DoA/KRF and the state-of-the-art DoA/KRF present similar accuracy, although the computational complexity of the proposed approach is considerably smaller than the state-of-the-art counterpart. Nonetheless, when comparing the performances of PAST–HOSVD/TDE and HOSVD/TDE it is observed that a small positioning error is produced. This difference relies on the fact that in the HOSVD-based approach a larger number of subspaces are tracked. On the other hand, on the computational complexity of the simulation, the reduction of the cost is more expressive.

The proposed methods reduce the cost, allowing best performance for applications that require real-time processing. Note that in this work only the additive noise and the multipath components are considered. The proposed techniques need to be adapted when taking into account propagation effects such as refraction in the ionosphere or troposphere. These and other related effects are considered as future work.

## Figures and Tables

**Table 1:** Numerical Complexity of the algorithms.

Algorithm	Complexity
HOSVD/TDE [10]	O(3(MKQ))
Proposed PAST–HOSVD/TDE	O(3(ML))
DoAKRF [11]	O(MKQ)
Proposed PAST–DoA/KRF	O(ML)

## Abbreviations

The following abbreviations are used in this paper:

GNSS	Global Navigation Satellite Systems
LOS	Line of Sight
NLOS	non-Line of Sight
SVD	Singular Value Decomposition
HOSVD	High Order Singular Value Decomposition
SCA	Safety-Critical Applications
LCA	Liability Critical Applications
PARAFAC	Parallel Factor Analysis
HOSVD/TDE	High Order Singular Value Decomposition based Time Delay Estimation
DOA/KRF	DoA/Khatri-Rao Factorization
SECSI	Semi-algebraic framework for approximate Canonical Polyadic Decompositions via
	Simultaneous Matrix Diagonalizations
CPD-GEVD	Canonical Polyadic Decomposition by Generalized Eigenvalue Decomposition
PAST	Projection Approximation Subspace Tracking
FAPI	Fast Approximated Power Iteration
FDPM	Fast Data Projection Method
FBA	Forward-Backward Averaging
ESPS	Expanded Spatial Smoothing
ESPRIT	Estimation of Signal Parameters via Rotational Invariance Techniques
KRF	Khatri-Rao factorization
DLR	German Aerospace Center
SMR	Signal to Multipath Ratio
MC	Monte Carlo
LPA	Largest Principal Angle

## Appendix A

This appendix contains the pseudo-code for DoA/KRF [11] which can be consulted in Algorithm A1.

**Algorithm A1:** DoA/KRF Time-delay estimation [11].
1. ESPRIT [37] section: Estimate the angle of arrival of the *L* received signals. Initialization: 1.1 – Define the selection matrices $J_{1}$ and $J_{2}$. $J_{1}=\left[I_{M}\;\;0_{M\times 1}\right]$ $J_{2}=\left[0_{M\times 1}\;\;I_{M}\right]$ Obtain an estimation for the signal subspace. ${\hat{U}}_{s}=\mathrm{EVD}\left(R_{xx}\right)$ Solve the shift invariance equation to obtain an estimation for Ψ. $\hat{\Psi }={\left(J_{1}{\hat{U}}_{s}\right)}^{+}J_{2}{\hat{U}}_{s}$ Obtain an estimation for the spatial frequencies ${\hat{\mu }}_{\ell }$. $\left\{{\hat{\mu }}_{\ell }\right\}=\mathrm{diag}\left\{\mathrm{EIG}\left(\hat{\Psi }\right)\right\}$ Find the corresponding angles of arrival $\left\{{\hat{\theta }}_{\ell }\right\}$. $\left\{{\hat{\theta }}_{\ell }\right\}=\arcsin\left(-\frac{1}{2\pi }\left\{{\hat{\mu }}_{\ell }\right\}\right)$ 2. Obtain each steering vector $\hat{a}\left(\theta_{\ell }\right)$ related to the array steering factor matrix. for ℓ=1,…,L do: $\hat{a}\left({\hat{\theta }}_{\ell }\right)=\left[\begin{array}{cccc} 1 & e^{j{\hat{\theta }}_{\ell }} & \cdots & e^{j\left(m-1\right){\hat{\theta }}_{\ell }} \end{array}\right]$ end 3. Rebuild the array steering factor matrix. $\hat{A}\left(\theta_{\ell }\right)={\left[\begin{array}{ccc} a(\theta_{1}) & \cdots & a(\theta_{\ell }) \end{array}\right]}^{T}$ 4. Obtain an estimation for the Khatri-Rao product. $\left(\Gamma^{T}⋄{\left({\mathrm{CQ}}_{\omega }^{\left(d\right)}\right)}^{T}\right)=\mathrm{transpose}\left\{{\hat{A}}^{+}{\left[Y\right]}_{\left(3\right)}\right\}$ 5. Estimate the remaining factor matrices by means of the Khatri-Rao factorization. for ℓ=1,…,Ldo: $\underset{Q\times K}{\mathrm{unvec}}\left\{{\left(\Gamma^{T}⋄{\left({\mathrm{CQ}}_{\omega }^{\left(d\right)}\right)}^{T}\right)}_{\left(:\;,\;\ell \right)}\right\}={\left({\mathrm{CQ}}_{\omega }^{\left(d\right)}\right)}_{\left(:\;,\;\ell \right)}^{T}{\left(\Gamma_{\left(:\;,\;\ell \right)}^{T}\right)}^{T}$ $\mathrm{SVD}\left\{{\left({\mathrm{CQ}}_{\omega }^{\left(d\right)}\right)}_{\left(:\;,\;\ell \right)}^{T}{\left(\Gamma_{\left(:\;,\;\ell \right)}^{T}\right)}^{T}\right\}=u_{\ell }\;\sigma_{\ell }\;v_{\ell }^{H}$ ${\hat{\Gamma }}_{\left(:\;,\;\ell \right)}^{T}=v_{{}_{\ell }}^{*}{\sqrt{\sigma }}_{\ell }$ ${\left(C{\hat{Q}}_{\omega }\right)}_{\left(:\;,\;\ell \right)}^{T}=u_{\ell }{\sqrt{\sigma }}_{\ell }$ end 6. With ${\hat{\Gamma }}^{T}$ and ${\left(C{\hat{Q}}_{\omega }^{\left(d\right)}\right)}^{T}$ perform the time-delay estimation. for ℓ=1,…,L do: $\left\{\ell_{\mathrm{index}}\right\}=\left|\right|{\tilde{\Gamma }}_{\left(:\;,\;\ell \right)}^{T}{\left|\right|}_{2}$ end $\ell_{\mathrm{LOS}}=\max_{\ell }\left\{\ell_{\mathrm{index}}\right\}$ ${\hat{c}}_{\mathrm{LOS}}={\left(C{\hat{Q}}_{\omega }\right)}_{\left(:\;,\;\ell_{\mathrm{LOS}}\right)}^{T}$ $q_{\mathrm{DoA}/\mathrm{KRF}}={\hat{c}}_{\mathrm{LOS}}\;\Sigma \;V^{H}$ ${\hat{\tau }}_{LOS}=\underset{\tau }{\arg\;\max}\left\{F\left(\tau \right)\right\}$
